# Advantages and limitations for users of double pit pour-flush latrines: a qualitative study in rural Bangladesh

**DOI:** 10.1186/s12889-017-4412-7

**Published:** 2017-05-25

**Authors:** Faruqe Hussain, Thomas Clasen, Shahinoor Akter, Victoria Bawel, Stephen P. Luby, Elli Leontsini, Leanne Unicomb, Milan Kanti Barua, Brittany Thomas, Peter J. Winch

**Affiliations:** 1 International Centre for Diarrhoeal Disease Research, Bangladesh (icddr,b), Dhaka, Bangladesh; 2 Rollins School of Public Health, Emory University, 1518 Clifton Road, NE, Atlanta, GA 30322 USA; 3 Stanford University, Stanford, California, USA; 4 BRAC WASH Programme, BRAC Centre, 75 Mohakhali, Dhaka, Bangladesh; 5 Department of International Health, Johns Hopkins Bloomberg School of Public Health, Baltimore, MD USA; 6 Programme for Emerging Infections, Infectious Diseases Division, icddr,b, 68 Shaheed Tajuddin Ahmed Sarani, Mohakhali, Dhaka, 1212 Bangladesh

**Keywords:** Feasibility, Double pit pour-flush latrine, Rural Bangladesh, Sanitation System

## Abstract

**Background:**

In rural Bangladesh, India and elsewhere, pour-flush pit latrines are the most common sanitation system. When a single pit latrine becomes full, users must empty it themselves and risk exposure to fresh feces, pay an emptying service to remove pit contents or build a new latrine. Double pit pour-flush latrines may serve as a long-term sanitation option including high water table areas because the pits do not need to be emptied immediately and the excreta decomposes into reusable soil.

**Methods:**

Double pit pour-flush latrines were implemented in rural Bangladesh for ‘hardcore poor’ households by a national NGO, BRAC. We conducted interviews, focus groups, and spot checks in two low-income, rural areas of Bangladesh to explore the advantages and limitations of using double pit latrines compared to single pit latrines.

**Results:**

The rural households accepted the double pit pour-flush latrine model and considered it feasible to use and maintain. This latrine design increased accessibility of a sanitation facility for these low-income residents and provided privacy, convenience and comfort, compared to open defecation. Although a double pit latrine is more costly and requires more space than a single pit latrine the households perceived this sanitation system to save resources, because households did not need to hire service workers to empty pits or remove decomposed contents themselves. In addition, the excreta decomposition process produced a reusable soil product that some households used in homestead gardening. The durability of the latrine superstructures was a problem, as most of the bamboo-pole superstructure broke after 6–18 months of use.

**Conclusions:**

Double pit pour-flush latrines are a long-term improved sanitation option that offers users several important advantages over single pit pour-flush latrines like in rural Bangladesh which can also be used in areas with high water table. Further research can provide an understanding of the comparative health impacts and effectiveness of the model in preventing human excreta from entering the environment.

## Background

An ongoing debate exists about the role of latrines alone or in combination sanitation interventions, in improving sanitation and health outcomes [1–9]. Recent evaluations of latrine installation have not detected the anticipated impacts on health or nutrition [2, 3]. However, some studies have found that sanitation upgrades led to improvements in child growth [1, 6]. These mixed results may be due to inadequate latrine coverage, use and maintenance, in addition to poor systems for managing fecal sludge [10]. This study explores the potential role of a double pit pour-flush latrine design in improving latrine acceptance and use and facilitating safe removal and treatment or reuse of fecal sludge.

Approximately 1.8 billion people from low-income countries use pit latrines as their usual form of sanitation [11]. Pit latrines are an improved sanitation option that have the potential to separate human excreta from the surrounding household environment and reduce the transmission of fecal-oral transmitted diseases [9]. In rural Bangladesh, it is common for households to use pour-flush pit latrines [12]. Single pit latrines comprise a latrine pan and pit, which are separated by a water seal that helps reduce odors and fly contact with feces [13]. Due to the high water table in most areas, pits are shallow (1–1.5 m) and fill after 12–24 months of use.

When single pit latrines fill, a new latrine should be built, or the pit emptied [14]. Single pit latrine users must spend money to buy new latrine components or hire pit emptying workers. Manually emptying fresh excreta presents a number of health risks, including exposure to helminth eggs [15]. The emptying process also has the potential to contaminate the household environment and surrounding areas where the fresh excreta are released.

**Fig. 1:**
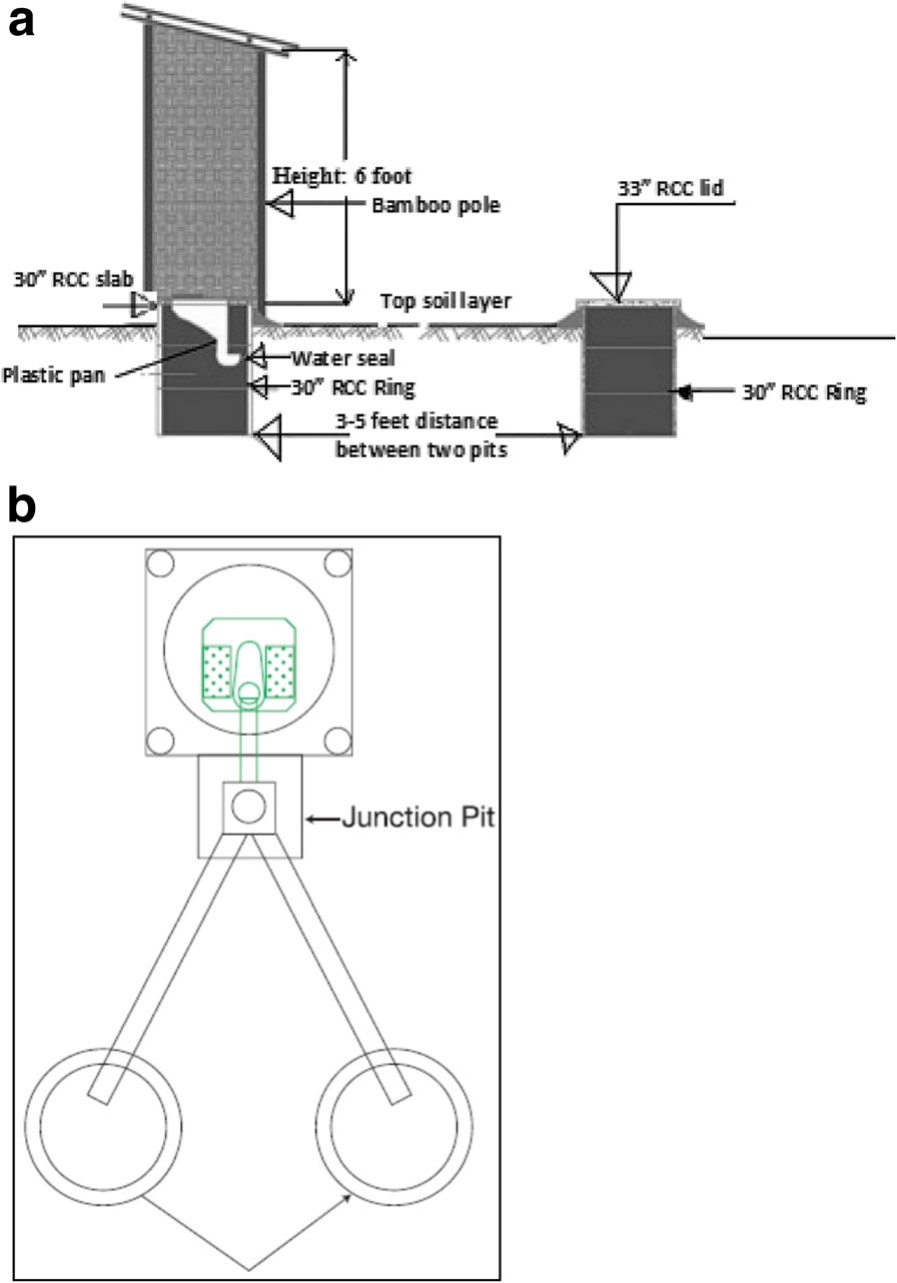
**a** double pit latrine layout (on-set model). **b** double pit latrine layout (off-set model)

Double pit latrine systems address many of the problems inherent in the single pit latrine design. When the first pit fills, users divert the waste stream to the second pit and allow the contents of the first pit to decompose [16]. Users thus move the superstructure from one pit to another (system evaluated in this study), or redirect the tube or pipe leading away from the toilet, from the full pit toward the empty pit (offset double pit latrine) (Fig. 1a﻿﻿﻿). Pathogens, including helminth eggs, are greatly reduced in the decomposition process [17–19]. After the excreta in the first pit decompose, the excreta can be safely emptied by household members and used as soil amendment in homestead gardening [13, 16, 20]. The decomposition process usually takes 12–18 months, and during this time, household members use the second pit [13].

Given the limitations of single pit latrines and the health hazards associated with emptying fresh excreta, the double pit pour-flush latrine system may greatly improve sanitation in areas like Bangladesh. Householders may be less resistant to use latrines that offer a feasible solution (and a beneficial byproduct) to pit emptying. Despite these benefits, there are some barriers to scaling up double pit pour-flush latrines. First, they are more expensive than single pit latrines (detailed below). Second, they require sufficient space for the second pit, which is often unavailable in higher density settings even in rural villages. An offset double pit latrine where the superstructure remains in place and the waste stream is diverted to the second pit also requires careful construction to ensure proper flow. These barriers may lead governments and NGOs to hesitate to invest in them.

Double pit pour-flush latrines have been implemented in Bangladesh by BRAC since 2006 in different regions of Bangladesh under their WASH (Water, Sanitation and Hygiene) Programme [21]. We collaborated with BRAC to conduct a qualitative assessment among households from the BRAC-WASH Programme intervention. We explored the advantages and limitations of using double pit pour-flush latrines compared to single pit latrines in low-income, rural areas of Bangladesh, from the perspective of households where the latrines were installed.

## Methods

### Study site and population

This qualitative study was conducted from October 2010 to April 2011 in Kishoreganj Sadar and Katiadi, rural sub-districts of Kishoreganj District in northeastern Bangladesh. These two sub-districts within the BRAC WASH Programme implementation area share similar socio-economic and geographical characteristics. The study was conducted amongst government-designated ‘hardcore poor’ households in these communities. The Pro-Poor Strategy for Water and Sanitation Sector in Bangladesh has defined ‘hardcore poor’ as: “landless households, pavement dwellers/homeless, the main-earning person or head of family is a day laborer, owning less than 0.202 hectares (50 decimal) of agriculture land or residing in a rented premise less than 200 square feet, and, having no fixed source of income and household is headed by a disabled person or female or old aged (65+ years) persons” [22]. BRAC staff formed a Village WASH (Water, Sanitation and Hygiene) Committee comprised of local government and community leader representatives to monitor the project’s implementation.

### Study design

To understand community acceptance, attitudes and experiences with use of the double pit pour-flush latrine, we conducted focus group discussions and in-depth interviews with adult users, guided by the Integrated Behavioral Model for Water, Sanitation and Hygiene (IBM-WASH) [23]. We also captured comments made by the neighbors during data collection. We observed the physical condition of the latrines and pattern of latrine use in the study households. To capture a broad range of user experiences, we interviewed households of various family sizes and geographical locations, as well as households headed by women and men, to consider factors influencing latrine use and maintenance beyond socio-economic influence.

### Sampling

Households were selected from the BRAC’s WASH Programme participants. These participants were classified as ‘hardcore poor’ population and being hardcore poor was an eligibility requirement for receiving a double pit latrine from BRAC. In general, each eligible household received a double pit pour-flush latrine and had been using the latrine regularly.

BRAC WASH Programme had preset criteria for eligibility that were not assigned by this study. Participating households were eligible if they were ‘hardcore poor’, which BRAC had determined using a survey that collected socio-economic data from household members. In addition, following BRAC’s criteria participating households (i) previously lacked improved sanitation facilities or access to improved latrines and practiced open defecation, (ii) received and had been using a double pit pour-flush latrine for at least 3–6 months, (iii) shared the cost of transporting the latrine’s components, and (iv) provided the labor to dig two pits and install the components. To provide ownership and increase value of the latrine, BRAC motivated the participants to share the cost of transport which was a basic criterion for a participating household.

We conducted in depth interviews and focus group discussions with households during October 2010 to April 2011. We interviewed members of 18 households, which had been using the double pit pour-flush latrine system for varying lengths of time. We expected that at least five households from each stage should cover the latrine life-cycle, and based on previous experiences, we expected that 20 households would be required for data saturation. Finally, 18 interviews covered the full latrine life-cycle, and we found data saturation. We included more households in the first and second stage to understand acceptability, implementation, patterns of use and maintenance including pit switching. We led five focus group discussions with households represented a low income levels within mixed income neighborhoods. In each focus group, eight to nine members participated. The groups were assigned by gender (male, female and mixed) and duration of use (less than 6 month and more than 6 months) of the double pit latrine.

BRAC had implemented double pit pour-flush latrine in different phases. We aimed to obtain feedback and experience from the users on the complete ‘life-cycle’ of the latrine. Therefore, households were intentionally selected to represent a continuum of users in the latrine ‘life-cycle’ (Table 1). During selection, household size, geographical location, and gender of the household head were also considered. Based on the duration of use of the latrine we classified households into three stages. Stage 1 households were defined as those using the latrine for three to six months prior to being interviewed. Stage 2 households were those who had recently switched their superstructure and begun using the second pit. Stage 3 households had emptied their first pit and ﻿s﻿﻿﻿﻿witched pits multiple times (Table 1).

**Table 1:** Distribution of participating households by latrine life-cycle stage

Stage	Approximate duration of use (months)	No. of households	Description of life-cycle stage
1	3–6	9	Install latrine and use first pit
2	7–12	6	Switch to second pit and use second pit
3	13–18+	3	Recycle decomposed excreta and switch back to first pit (or any subsequent switch)

### Double pit pour-flush latrine model and implementation

The double pit pour-flush latrine design included several components that users could easily assemble on their own. The components could be switched between two separate pits, placed three to five feet in distance from each other without any piped connection (Fig. 1b). The latrine components included six concrete rings (three rings for each pit), a concrete latrine lid, a concrete squatting slab, a plastic pan, a plastic water seal, four bamboo stability poles, three fences, a roof and a door, all made of bamboo. The concrete rings lined the pit and were 0.3 m tall by 0.8 m in inner diameter. The average volume of a three ring pit was 0.416m^3^. The latrine lid covered the pit not in use. The bamboo poles were used to construct the superstructure with the fencing on three sides, the door, and the roof.

The physical components of the latrine model cost approximately $28 USD (based on April 10, 2011 exchange rate; 1 USD = 72.7 BDT). This compared to an estimated $20 for a single pit latrine, a difference of 40%. In this case, BRAC provided the components free of cost. The transportation of supplies, construction of pits, and assembly of pit components varied in cost from an estimated $11–14 USD depending on the location of production centers, households and road conditions. The households provided all the cost for transportation and the labor for assembly and installation.

### Data analysis

We categorized the interview and group responses based on themes including: feasibility and patterns of latrine use, operation and maintenance, perceived benefits and perceived barriers of using a double pit pour-flush latrine. Drawing from the three dimensions in the IBM-WASH model, contextual, technological, and psychosocial factors were identified which influenced the feasibility and acceptability of double pit pour-flush latrine use and maintenance at the household and community level [24].

The IBM-WASH model is a social-ecological model. It differs from other social-ecological models in having a level for the household and a level for habit formation (habitual) due to the importance of household-level factors and habit formation in WASH behaviors. It has separate columns or dimensions for contextual, psychosocial and technology factors. In this case, the context was the social and physical environment for the latrines, and psychosocial factors included knowledge, social norms and disgust. Technology factors were specific to the latrine design which affected its use and maintenance [24].

## Results

Of the 18 households with latrines at various stages of use (Table 1), nine households were still using the first pit (Stage 1). Six households had filled the first pit and switched to using the second pit (Stage 2). Three households had utilized composted excreta from the first pit and switched pits multiple times (Stage 3). The heads of households included rickshaw pullers (6), shopkeepers (4), day laborers (3), small scale farmers (3), a fisherman (1), and a differently able man (1) (Table 2).

**Table 2:** Participant household characteristics

Characteristics	*N* = 18
Occupation of household heads	
Farmer/ Sharecropper	3
Wage labor	3
Van/Rickshaw puller	6
Fisherman	1
Shopkeeper	4
Disabled/aged	1
No. of users per double pit latrine	
≤ 6	10
7–10	6
≥ 11	2

### Double pour-flush pit latrine utilization

Prior to the study and acquisition of a latrine, most study participants had reported that they defecated in the open; others shared a latrine with neighbors. Upon installation and use of the new latrines, focus group participants noticed a reduction in flies, mosquitoes, and bad odors in the household environment. Households perceived an increase in environmental cleanliness with the installation of a double pit pour-flush latrine. None of the latrines had visible feces around the latrine pits. The majority of the latrine pans appeared clean (15/18), and there was no odor (15/18). One of the participants from the focus groups and owner of a latrine said,

“Previously we would defecate in the open which smelled bad. You (referring to researchers) wouldn’t be able to stay here if you would have come earlier (before installing this latrine) (female, 55, married, homemaker)”.

The new latrines also provided users with greater privacy, convenience, comfort, and social status compared to practicing open defecation or using unimproved latrines. Users appreciated that there were no snakes, wild animals and insects around the latrine. One woman noted:

“Previously I used to defecate in (the) open, in the bush or in abandoned or waste ponds. I became frightened (startled) and I stood up when I felt that someone may notice I am defecating in open. Now I don’t have that fear (female, 37, married, homemaker)”.

Prior to latrine ownership, households could not provide latrine facilities to guests. Participants reported that after receiving latrines, their social status improved, and they no longer felt embarrassed when relatives or guests visited. One of the respondents said,

“Lacking a latrine we had to request our neighbors to allow latrine access to our visitors and guests. But now I am happy that I have an improved latrine and our relatives also appreciate it (female, 32, married, homemaker)”.

Many households noted latrines were easy to construct, but the majority of users (12/18) noted problems with the latrine superstructure. Household members reported that within six months of latrine use, parts of the superstructure started to deteriorate. The bamboo poles used to stabilize the fences began to break and decay so that fences no longer maintained privacy for users. Five households reported that their superstructure broke when moving it to the second pit. Our field team observed fences and doors were broken in some of the latrines (Table 3). To build a replacement structure, participants had to purchase new superstructure materials. Three households mentioned that low-income households lack sufficient space in homesteads and the double pit pour-flush model requires more space than a single pit pour-flush model. Despite these limitations, our field team observed that all participant households had functional latrines with signs of use.

**Table 3:** Spot checks of double pit latrine conditions

Findings	*N* = 18
Latrine installation/no pit switch	9
Pit switched/shifted at least once	9
Pit switched/shifted more than once	3
Latrine functional and in use	18
No odor	16
Latrine pan clean	15
No feces around the latrine pits	18
Water seal functional (water in pot)	18
No broken pit liner	18
Second pit secure	18
Superstructure (same model)	18
Good condition	2
Broken	12
Changed	4

### Pit filling and switching

Household members were able to determine when the pit became full by using their own monitoring methods. Sound produced by water and feces dropping into the pit was mentioned as a common method used to approximate the remaining empty space in the pit. Household members said that they also lifted the concrete slab to determine the remaining space.

Participants appreciated the ease in switching pits, as the process required only physical labor to dismantle, move and set up the superstructure over the second pit. Many households (12/18) were able to switch between pits without facing difficulties. Pit shifting was not arduous, and women performed this task in some households. One of the users said:

“I faced no problems to move the superstructure and swap between slab and lid. My son helped me along with my younger brother. We three people switched the pit which didn't cost even a Taka (female, 35, married, homemaker).”

Most households switched pits in a timely manner when the first pit became full. In most cases, households did not have feces overflowing from the latrine pan or pit. A small number (2/18) of households mentioned that they delayed pit switching and continued to use pits that were almost completely filled. As a result, the feces layer rose and submerged the latrine pan in excreta. After the slab was moved from the filled pit, fresh excreta were exposed. These households noted that they were disgusted with the visible fresh feces, which produced bad odors. One participant said,

“It was only disgusting to remove the slab from the full pit due to the odor. To continue latrine use we had nothing to do but switch pit within the existing circumstances (female, 34, married, homemaker).”

Users reported pits could fill before the contents of the previous pit had decomposed. Furthermore, pits could fill during heavy rainfall, which led users to either switch pits or to continue using the latrine after it was full. Areas with high water tables must have shallow pit depths, and some large households filled their pit within six months because of the shallow pit depth. One of the participants said,

“Within 6 months my latrine pit filled up. We have 8 family members and relatives visit us frequently which hastened the filling (male, 42, married, farmer)”.

The pit depth problem also related to concerns about the water seal in the latrine. Household members reported that greater amounts of water were required for flushing a latrine with a water seal, and this added water may contribute to earlier pit filling. Many of the participants stated that they had previously removed the water seal because they did not understand the benefits of the device; BRAC team members had visited the households early in the intervention to explain that the water seal prevented flies and mosquitoes from accessing the excreta and reduced bad odor and to encourage them to re-install the water seals. When our field team visited households later in the intervention, we found that water seals were functional, pit liner was in good condition and the second pit was secured in the latrines of all 18 study households (Table 3).

### Composting excreta and pit emptying

**Fig. 2:**
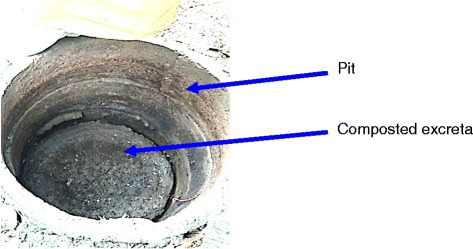
Composted excreta inside the pit

For stage 3 participants, the excreta in the first filled pit were left to decompose while the second pit was in use. They said that during the decomposition process, the surrounding soil absorbed water from the excreta, and the decline in the liquid level was visible. Approximately one year after pit switching, the contents of the pit reduced in volume, producing a dry, odorless soil product. We could directly observe this in one of the pits (Fig. 2).

The decomposition process resulted in savings in several ways. Participants mentioned that with a single pit latrine model, the pit must be emptied by hiring a pit emptying service or a new latrine must be constructed; that the double pit pour-flush latrine model allowed households to empty composted soil themselves and reuse latrine materials with the alternating pit system. The savings were estimated at $ 4 USD. One user who recognized this cost saving said:

“My brother emptied the pit contents. A *methor* (pit emptying service worker) would have charged at least 200-400 Taka! (US $2.75-$5.50) (female, 46, separated, homemaker)”.

In addition, households could use the composted soil for homestead gardening, and some women in the study reported using it in their gardens. Many households appreciated the savings and benefits of the latrine model. One of the participants said,

“We could have used the contents but we do not have our own land for agricultural production. I used those for fruit plants within my courtyard area (female, 34, married, homemaker).”

## Discussion

### Latrine Benefits compared to open defecation

Compared to open defecation, single and double pit latrines (pour-flush model) share many putative benefits for households including increased convenience, privacy, safety, social status and reduced environmental contamination with fecal pathogens [23]. Households in our study also identified similar benefits of latrine use.

Prior to latrine installation, most household members practiced open defecation. Latrines provided household members with an enclosed space to defecate so that they did not have to walk to the fields and search for a private place. Household members in our study appreciated the convenience, comfort and privacy provided by a latrine.

In addition, households perceived an increase in social status with latrine ownership. Participating households could provide their guests access to a latrine. Other scholars have found that increased social standing associated with latrine ownership has influenced latrine adoption in rural Bangladesh communities. Guiteras et al. evaluated the spillover effects of a subsidized latrine distribution program in 380 rural Bangladesh communities and discovered that both subsidy recipients and households neighboring subsidy recipients had increased latrine ownership due to the program, representing a social multiplier effect [25]. Routray et al. also noted that high social networks influenced latrine ownership in rural Indian households [23].

For a sanitation system to be maintained, latrine components need to be sustainable. In our study, most households (12/18) had latrine superstructures which were broken; the bamboo poles supporting the external fences of the latrine were decaying. Problems with the superstructure design could limit the continued use of double pit latrines over the longer term. Therefore, the materials of the superstructure should be improved to withstand long-term use and pit switching.

### Specific benefits of double pit latrines

The specific benefits of double pit technology mentioned in the literature, include: ability of the user to empty the pit, generation of a decomposed soil product, reduction of environmental contamination, and continued availability of a pit over the long term [9, 16, 26]. Study participants reported three of these four benefits, namely the ability of the user to empty the pit, the generation of a decomposed soil product, and the continued availability of the pit for future use over a complete “latrine life-cycle”. However, the perceived savings from self-emptying the pit were additional benefits that household users described.

With a single pit latrine, households are faced with five options upon pit filling. First, they can abandon the latrine and build a new latrine which has cost implications. Second, they can stop using the latrine and return to open defecation. Third, they can hire a pit emptying service, with its associated costs and health risks. Fourth, they can make a hole in the side of the pit (pit diversion), and finally, they can empty the pit with a pipe occasionally (flooding out). The last four options can contaminate the surrounding water bodies or land with the pit contents. In a study on improved sanitation in Dar es Salaam, the most common design was the traditional (single) pit latrine (88%). The study found that 28% of surveyed households had an emptying pipe to flood out pits [27]. The double pit pour-flush latrine technology assessed in our study circumvents these problems.

Furthermore, when single pits fill, their emptying may contaminate the surrounding environment with helminth eggs and other pathogens [15]. However, the composting process in double pit latrine significantly reduces fecal pathogens and the concentration of helminth eggs in excreta [17–19]. For these reasons, the single pit latrine is not a sustainable sanitation solution nor environmentally safe compared to the double pit latrine [11]. Ramani et al. describes the double pit latrine system as closing the “loop” of the latrine “life cycle” by recycling the excreta and reducing health and environmental risks of excreta disposal [26]. Households in our study appreciated this long-term technology, but they did not identify this lowered risk of contamination as a benefit. The ability to manually empty decomposed pit contents, as opposed to hiring a pit emptying service, led to perceived cost savings among our study households.

Proper and regular maintenance of double pit pour-flush latrines may produce compost with reduced pathogen levels and helminth eggs that can be used in agricultural fields or in gardening. Poorer households with no agricultural land may sell the compost to the farmers who realize the benefits.

### Implementing Double Pit Pour-flush Latrines

When implementing latrine sanitation programs, single and double pit systems (pour-flush model) are the two options. Government officials, donor organizations and NGOs commonly assume that double pit latrines are more expensive to install than single pit latrines. While this is true, the additional costs are modest (approximately $10) and households perceived considerable savings associated with double pit latrines, and these perceptions may influence their continued use and maintenance. These benefits offer strong support for implementing double pit latrines.

The bamboo poles easily decomposed, leading to the superstructure breaking during pit switching of on-set model (Fig. 1a) or continual use. A double pit model in which the pits are offset instead of being located directly under the superstructure and squatting slab so that the superstructure does not need to be moved (Fig. 1b) would encourage the construction of more durable superstructures and could prolong the lifespan of double pit latrine sanitation systems. This design, would, however, increase costs.

The benefits of the double pit pour-flush latrine system are limited by the shallow pit depth due to the high water tables in Bangladesh. Pits should be dug as deep as possible withinthe constraints of the high water table but not more than a man’s height considering worker’s safety. As the manual emptier must enter into the pit, health risks increase when pits are deeper than 1.5 m, [28, 29], where low oxygen and toxic gases such as ammonia and methane may be present. In addition, glass and metal pieces in the sludge can cause cuts [30]. It is important to educate household users about water tables rising and consequent faster pit filling during heavy rainfall. Each pit should be raised higher than the normal ground level so that rain water does not enter the pit. Since households tend to break the water seal to reduce the water volume needed for flushing so that the pit does not fill as quickly, the importance of the water seal in reducing environmental contamination and odors should also be emphasized to users. It is also important to emphasize shifting to a new pit before the existing one fills or overflows.

According to the IBM-WASH framework guiding this study [24], behavior change strategies and educational campaigns should pay attention to the technological dimension of sanitation interventions and teach households the details on how to use and maintain the double pit latrine system. Researchers should continue to monitor the lifespan of the double pit latrine system and environmental contamination risks of sanitation systems.

### Limitations

The limitations of the study included a specific geographic location and target population, and free latrine provision. In other geographical and high water table areas, users may experience different difficulties. The households in the BRAC intervention and our study were from two rural Bangladesh communities defined as ‘hardcore poor,’ a population that does not represent the larger rural community population.

## Conclusions

Low-income households in rural Bangladesh accepted and utilized subsidized double pit pour-flush latrines. The study reinforced the putative benefits of double pit latrines compared to single pit latrines including: the ability of the user to empty the pit, the generation of a decomposed soil product, and the implementation of a durable sanitation system. Study participants also noted that manual self-emptying led to savings by not needing to hire a pit emptying service and the further free utilization of the soil product. In areas with high water tables, the double pit pour-flush latrine offers many benefits as well.

Although double pit pour-flush latrines cost more than single pit pour-flush latrines, they are a superior sanitation system with a longer lifespan than single pit latrines. When implementing new sanitation systems, it is important to note and communicate the many benefits of double pit latrines compared to single pit latrines.

## Abbreviations

BRAC: A national NGO
IBM-WASH: Integrated Behavioral Model for Water, Sanitation and Hygiene
NGO: Non-governmental Organization
USD: United States Dollar
WASH: Water, Sanitation and Hygiene
